# Multi-omics analyses reveal the crosstalk between the circadian clock and the response to herbicide application in *Oryza sativa*

**DOI:** 10.3389/fpls.2023.1155258

**Published:** 2023-03-24

**Authors:** Ke Chen, Xiao Su, Haona Yang, Yajun Peng, Lamei Wu, Zhenghong Zhao, Tao Lin, Lianyang Bai, Lifeng Wang

**Affiliations:** ^1^ Longping Branch, College of Biology, Hunan University, Changsha, China; ^2^ Key Laboratory of Indica Rice Genetics and Breeding in the Middle and Lower Reaches of Yangtze River Valley, Ministry of Agriculture and Rural Affairs, Hunan Rice Research Institute, Hunan Academy of Agricultural Sciences, Changsha, China; ^3^ Huangpu Research Institute of Longping Agricultural Science and Technology, Guangzhou, China; ^4^ State Key Laboratory of Agrobiotechnology, Beijing Key Laboratory of Growth and Developmental Regulation for Protected Vegetable Crops, College of Horticulture, China Agricultural University, Beijing, China

**Keywords:** *Oryza sativa L.*, circadian clock, herbicides, RNA-seq, metabolome

## Abstract

Plants have evolved circadian clock systems that enable biological processes to occur in tandem with periodic changes in the environment. However, it is largely unknown whether crosstalk occurs between the circadian clock and the response to herbicide in rice. We identified 19 conserved rhythmic metabolites which were response to pesticide application and their metabolic abundance peaked mainly at ZT2 or ZT14-ZT18. We found a series of glyphosate, s-Metolachlor, fenclorim, metcamifen and GA3 response genes were expressed following stable circadian rhythms. In order to determine the patterns of their temporal expression, co-expression network analysis was done on 10,467 genes that were periodically expressed throughout a 24-hour period. Next, we identified 4,031 potential direct target genes of *OsCCA1* in using DAP-seq data for *OsCCA1*. Of these, 339, 22, 53, 53 and 63 genes showed a response to glyphosate, s-Metolachlor, fenclorim, metcamifen and GA3 application, respectively. And they were mainly phased from dusk to midnight. Interestingly, we identified significant *OsCCA1* binding peaks in the promoter regions of four herbicide resistance genes, including *OsCYP81A12*, *OsCYP81E22*, *OsCYP76C2*, and *OsCYP76C4*. Finally, we found that herbicide application could affects the expression of some of the central oscillator genes of the rice circadian clock. Here, we used multi-omics data to reveal the crosstalk between the circadian clock and herbicide response processes at the epigenomics, transcriptome, and metabolome levels in rice. This work will serve as a theoretical guide for identifying rhythmic herbicide targets, leading to the creation of new herbicides or the breeding of crops resistant to herbicides.

## 1 Introduction

Rice (*Oryza sativa L.*) is one of the most important crops and is a valuable food source for approximately 4 billion people worldwide (Sasaki and Burr, 2000). The use of pesticides has been increased exponentially in recent decades due to their excellent protective power (Maggi et al., 2019). Pesticides are biologically active xenobiotic compounds that have at least one specific target site and the inhibition of target site activity is the main mode of action of pesticides. Although the application of pesticides is based on an evaluation of their visible phytotoxicity to non-target crops, there are likely to be many non-visual and subtle effects on the crops at the physiological, biochemical, and molecular levels (Banerjee et al., 2001; Bhatnagar-Mathur et al., 2008; Cheng et al., 2012; Ding et al., 2014).

To adapt to the periodic changes in light, temperature, and other environmental factors resulting from the rotation of the Earth, organisms have evolved complex circadian clock systems that enable their physiological processes to occur in accordance with periodic changes in environmental factors (Edgar et al., 2012). The circadian clock not only facilitates the adjustment of circadian oscillations in response to the light/dark cycle and maintenance of regional adaptability in plants but also participates in regulating various biotic/abiotic stress responses. In *Arabidopsis thaliana*, CIRCADIAN CLOCK ASSOCIATED 1 (*CCA1*), the central circadian oscillator, confers heterosis for defense against bacteria in hybrids without growth vigor penalties, and significantly enhances the growth heterosis of hybrids infected with pathogens (Yang et al., 2021). Mutations in the central clock component LUX ARRHYTHMO (*LUX*) disrupt jasmonic acid- and salicylic acid-mediated defense signaling, which results in compromised disease resistance (Zhang et al., 2019). In addition, Arabidopsis GIGANTEA (*GI*) affects adaptability to salt stress, through the Salt Overly Sensitive 2 (*SOS2*)-mediated SOS-signaling pathway (Kim et al., 2013). In rice, the central circadian oscillator component *OsCCA1* regulates tolerance to salt, drought, and osmotic stresses, by mediating the ABA signaling pathway (Wei et al., 2022). *OsPRR37* endows rice with tolerance to salt stress *via* the inhibition of transcription of high-affinity K+ transporters 2;1 (*OsHKT2;1*), thereby reducing the level of accumulated Na+ and reactive oxygen species (ROS) (Wei et al., 2021). Previous studies have shown that the application of herbicides at different times of the day results in different levels of efficiency in field, with the time of day being the second-most important factor (Martinson et al., 2002; Miller et al., 2003). Recently, it has also been shown that overexpression of the *Arabidopsis thaliana* central circadian oscillator *CCA1* and *TOC1* would affect the rhythmic effectiveness of glyphosate (Belbin et al., 2019). And several studies have been conducted to reveal the response of plants to pesticides and the circadian expression of plant endogenous genes and metabolites at the transcript and metabolite levels by sequencing. However, it remains largely unknown whether there is crosstalk between the circadian clock and the response to herbicide application at the transcriptional, metabolic, and other levels.

**Figure 1:**
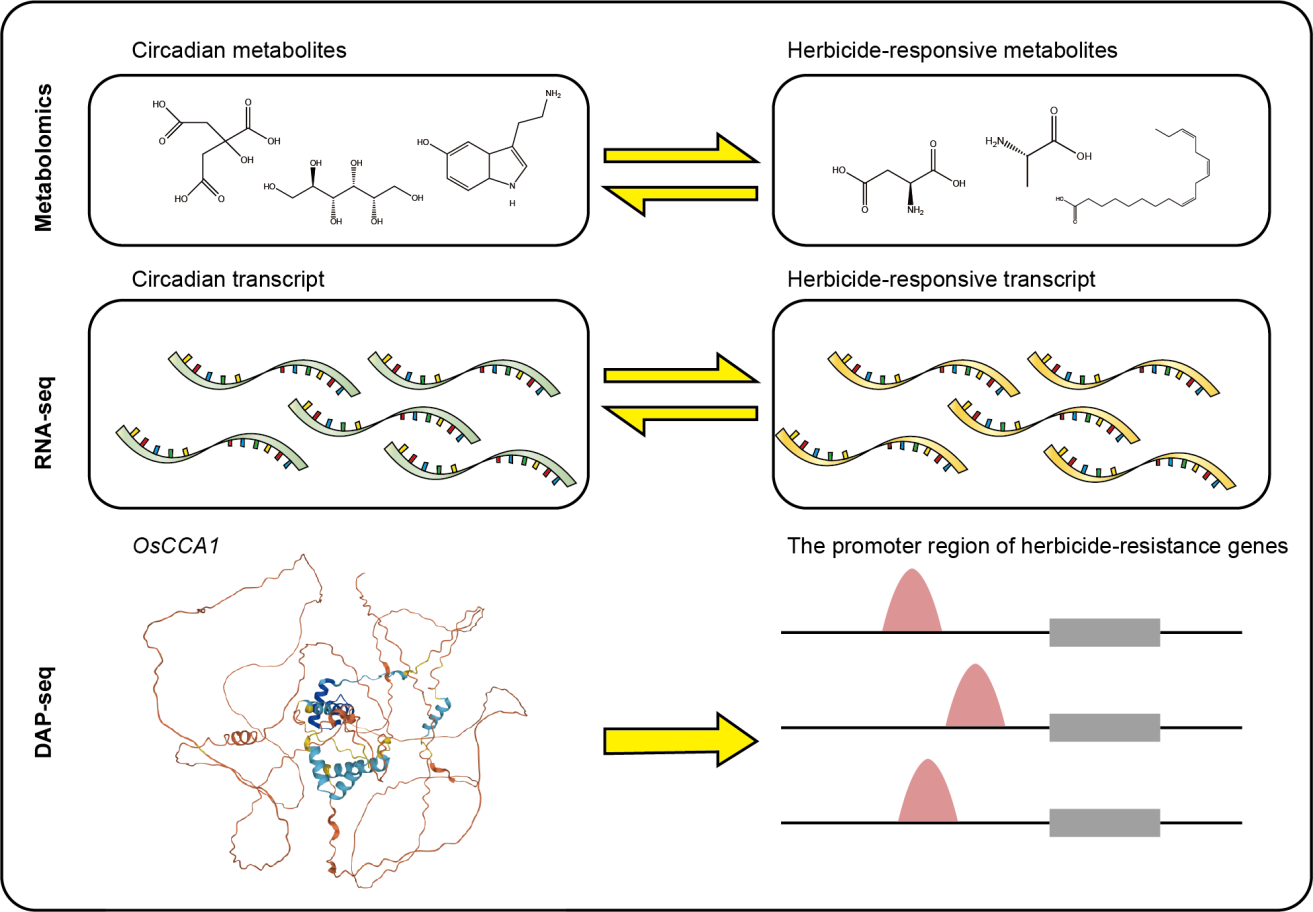
Schematic overview of the workflow and analysis pipeline in this study.

In this study, we first found 19 conserved rhythmic metabolites in response to pesticides application. To understand the molecular mechanisms of the crosstalk, we identified conserved and extremely conserved circadian genes in rice and analyzed the temporal expression patterns of these genes. In addition, we identified a series of conserved circadian genes could response to herbicides such as glyphosate, s-metolachlor and herbicide safeners such as fenclorim, metcamifen and GA3, and analyzed their expression patterns and functions. Further, we performed a co-expression network analysis to identify genes that were specifically highly expressed at each time. Next, we used the DAP-seq data of the rice central circadian oscillator *OsCCA1* to identify herbicide-responsive genes and herbicide resistance genes that may be directly regulated by *OsCCA1.* We found that some known herbicide non-target-site resistance genes might be directly regulated by *OsCCA1*. Further, we identified the effects of herbicide application on central circadian oscillators in rice. In this study, we used multi-omics data at the epigenomics, transcriptome, and metabolome levels to uncover the interaction between the circadian clock and the response to herbicide treatment in rice (**Figure 1**). This work will serve as a theoretical guide for identifying rhythmic herbicide targets, leading to the creation of new herbicides or the breeding of crops resistant to herbicides.

## 2 Results

### 2.1 A series of pesticide-responsive metabolites are rhythmic

**Figure 2:**
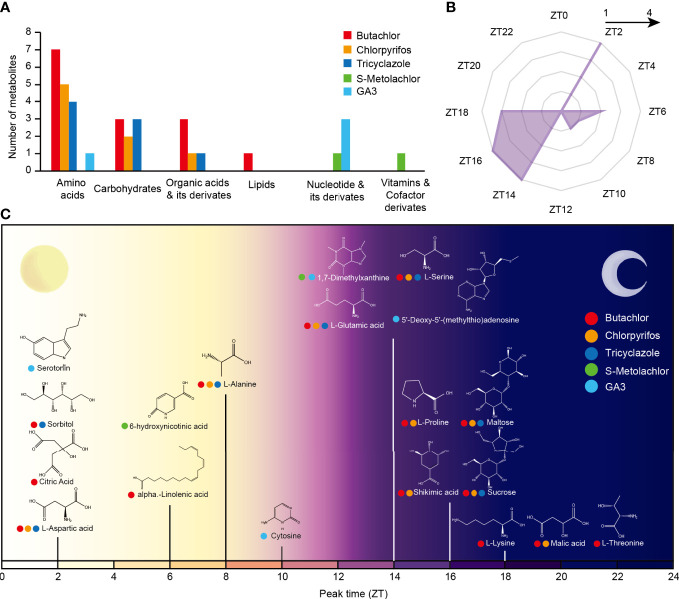
The crosstalk of rice circadian rhythm and pesticides response processes at metabolome level. **(A)** Number of various CRMs in response to the application of different pesticides. **(B)** Circadian phase distribution of pesticides responsive CRMs. The coordinates of each angle represent phases ZT0–ZT24. The inner to outer circles represent 1-5 metabolites reaching peak expression at that time point, respectively. **(C)** Time of peak accumulation of pesticides responsive CRMs. The x-axis represents the circadian phase of the metabolite. The different colors of the circles on the left of the metabolite’s name represent its response to different pesticides.

Previous study found the application of herbicide butachlor, insecticide chlorpyrifos, and fungicide tricyclazole affect the accumulation of many metabolites in rice (Liu and Zhu, 2020). And we found the application of the herbicide s-metolachlor and herbicide safener, also a plant hormone, gibberellin 3 (GA3) also showed an effect to rice (**Table S1**). In addition, a study found the accumulation of many metabolites in rice with stable circadian rhythms (known as circadian rhythmic metabolites - CRMs) has been observed (Zhou et al., 2022). However, whether these metabolites with circadian rhythms are induced by pesticides is still known, and much is currently unknown about the crosstalk between circadian rhythms and herbicide response in rice. Therefore, we used the metabolite data from the two studies mentioned above (**Table S2**) as well as metabolomic data measured by our team and identified 19 CRMs responded to pesticides application. We found that amino acids were the most frequent rhythmic metabolite clade to respond to pesticides, followed by carbohydrates and organic acids (**Figure 2A**).

We then analyzed the phase distribution of these 19 CRMs and found that these metabolites reached peak abundance both during the day and night. Daily-phased metabolites reached peak metabolic abundance mainly at dawn (Zeitgeber time 2 (ZT2, zeitgeber time relative to this experimental LD cycle, and in an LD cycle of 12h of light and 12h of darkness (LD 12:12) the time of lights on is denoted by ZT0, and the time of lights off by ZT12)), while night-phased metabolites reached peak metabolic abundance mainly before midnight (ZT14-ZT18) (**Figure 2B**).

Among the daily metabolites, the Serotonin, Sorbitol, Citric Acid and L-Aspartic acid were peaking in the morning (ZT2) and the 6-hydroxynicotinic acid, alpha.-Linolenic acid, L-Alanine and Cytosine were peaking in the afternoon (**Figure 2C**). And only Malic acid and L-Threonine reached peak expression after the midnight, while the rest peaked from dusk to midnight (**Figure 2C**). Importantly, 16 (84%) of the CRMs responded to the application of herbicide, which raises the possibility that the circadian clock is directly involved in a number of the metabolic pathways by which rice reacts to the application of herbicide.

### 2.2 A series of herbicide-responsive genes are rhythmic

**Figure 3:**
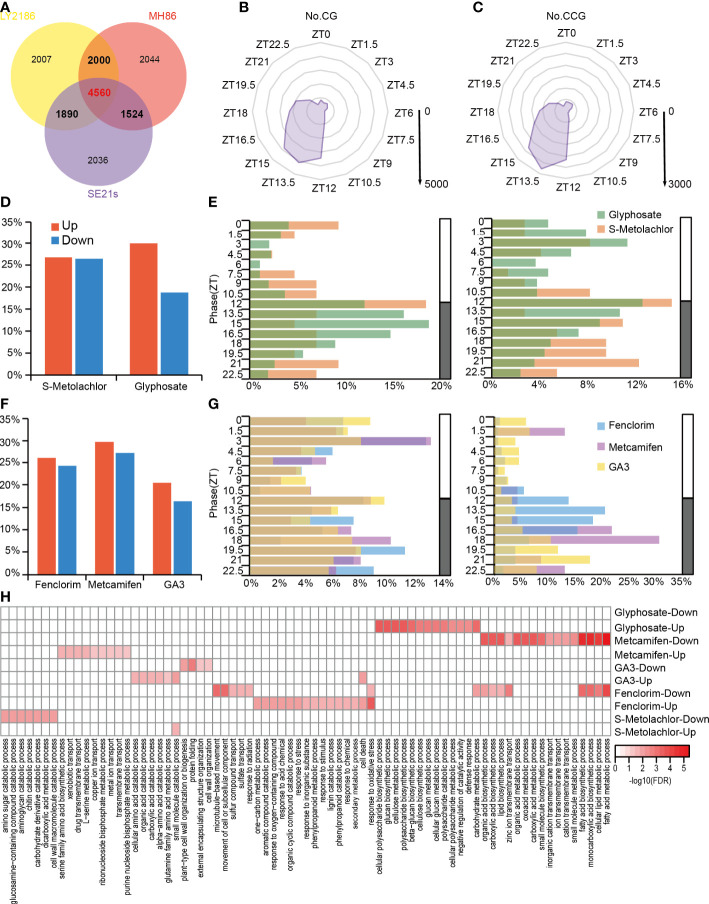
The crosstalk of rice circadian rhythm and herbicide response processes at transcriptome level. **(A)** Venn diagram indicating CGs, CCGs and specific genes is shown. Genes with a 24-hour stable circadian rhythm in at least two rice varieties are defined as CG (indicated by bold font); genes with a 24-hour stable circadian rhythm in all three rice varieties are defined as CCG (indicated by red bold font). **(B)** Phase distribution of the CGs. The coordinates of each angle represent phases ZT0–ZT24. The inner to outer circles represent 1,000-5,000 genes reaching peak expression at that time point, respectively. **(C)** Phase distribution of the CCGs. The coordinates of each angle represent phases ZT0–ZT24. The inner to outer circles represent 500-3,000 genes reaching peak expression at that time point, respectively. **(D)** The percentage of rhythmic herbicide-responsive genes in the total herbicide-responsive genes. **(E)** Phase distribution of rhythmic herbicide-responsive genes. The left panel is rhythmic herbicide-induced genes and right panel is rhythmic herbicide-suppressed genes. **(F)** The percentage of rhythmic herbicide safener-responsive genes in the total herbicide safener-responsive genes. **(G)** Phase distribution of rhythmic herbicide safener-responsive genes. Left panel is rhythmic herbicide safener-induced genes and the right panel is rhythmic herbicide safener-suppressed genes. **(H)** Heatmap of GO enrichment analysis of rhythmic herbicide-responsive genes.

To investigate the crosstalk between the circadian clock and herbicide application in rice, we identified circadian rhythm genes in three rice varieties in using the previous published RNA-Sequencing data (**Table S2**) (Li et al., 2021), and defined genes expressed by 24 hours period in at least two varieties as circadian genes (CGs) and genes expressed by 24 hours period in all three rice varieties as conserved circadian genes (CCGs). Totally, we identified 9,974 CGs and 4,560 CCGs (**Figure 3A**), and found that most CGs and CCGs were phased from dusk to midnight (ZT12-ZT15) (**Figures 3B, C**). Next, we performed gene ontology (GO) enrichment analysis of CGs (**Figure S1**) and CCGs (**Figure S2**), found that both were enriched in genes related to nitrogen synthesis and metabolism as well as small molecule metabolism. This indicated that rhythmic genes, which are conserved in many rice varieties, are mainly involved in nutrient uptake metabolism and plant growth metabolic processes.

Next, we identified circadian rhythmic genes that were influenced by herbicides and herbicide safeners application including glyphosate (**Table S2**) (Zhai et al., 2020), s-metolachlor, fenclorim (**Table S2**) (Hu et al., 2020), metcamifen (**Table S2**) and GA3, and found 1,069 glyphosate-responsive genes (**Table S3**), 51 s-metolachlor-responsive genes (**Table S4**), 182 fenclorim-responsive genes (**Table S5**), 122 metcamifen-responsive genes (**Table S6**) and 183 GA3-responsive genes that exhibited oscillations under light-dark cycles (**Table S7**). In other words, we found that 16.39%-29.82% of herbicide-responsive genes had stable circadian rhythms, with the highest percentage of s-metolachlor-induced genes and the lowest percentage of metcamifen-suppressed genes (**Figures 3D, F**).

For the rhythmic herbicide-induced genes, we found that the phases of s-metolachlor-induced genes showed a scattered distribution throughout the day, while those of 61.86% glyphosate-induced rhythmic genes reached peak abundance levels from dusk to midnight (ZT12-ZT16.5). As for the rhythmic herbicide-suppressed genes, we found that s-metolachlor-suppressed genes were mainly expressed at night, while glyphosate-suppressed genes had the most genes with peak expression around ZT3 and ZT12, respectively (**Figure 3E**). For the rhythmic herbicide safener-induced genes, we found that the phase of fenclorim-induced genes and GA3-induced genes showed a scattered distribution pattern throughout the day, the metcamifen-induced genes were mainly concentrated their expression peak at ZT3 and all night. And for the rhythmic herbicide safener-suppressed genes, we found 69.73% of fenclorim-suppressed rhythmic genes reached peak abundance from dusk to midnight (ZT12-ZT16.5), the GA3 and metcamifen-suppressed rhythmic genes more peaking in the ZT18-ZT22.5 and ZT16.5-ZT18, respectively (**Figure 3G**).

To investigate the functions possessed by rhythmic herbicide response genes, we performed GO functional enrichment analysis of rhythmic herbicide-induced or suppressed genes. For herbicides application, we found that rhythmic glyphosate-induced genes are mainly involved in glucan biosynthesis, metabolism and other processes; rhythmic S-Metolachlor-induced genes are mainly involved in small molecule catalytic processes while suppressed genes are mainly involved in carbohydrate biosynthesis processes. For herbicide safeners application, we noticed the rhythmic Fenclorim-induced genes mainly involved in stress response processes, while the suppressed genes are mainly involved in the carbohydrate synthesis and metabolism of carbohydrate such as fatty acids; the rhythmic Metcamifen-induced genes are mainly involved in some transmembrane transport processes while the suppressed genes are also involved in carbohydrate synthesis and metabolism; the GA3-induced genes are mainly involved in some acid catalytic processes while the suppressed genes are mainly involved in some cellular organization processes. This suggests that for different herbicides, the effect of circadian rhythms on their response processes may be different (**Figure 3H**).

### 2.3 Temporal expression pattern of rhythmic herbicide-responsive genes

**Figure 4:**
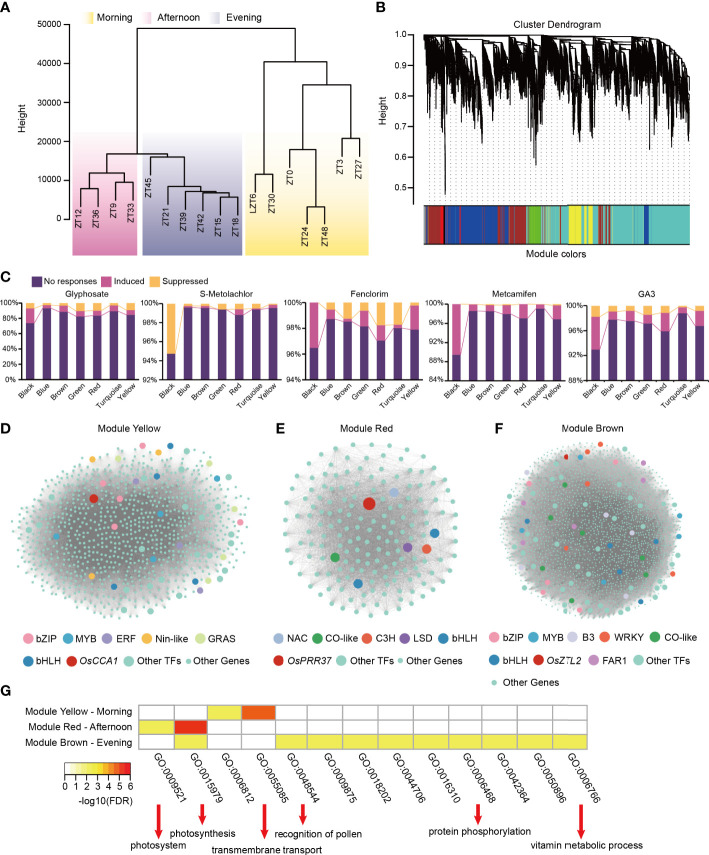
Co-expression analyses of 24h periods circadian rhythm genes. **(A)** Hierarchical clustering dendrogram showing samples grouping into morning, afternoon and evening clusters. **(B)** Dendrogram showing transcript co-expression modules (clusters) identified by WGCNA across different times. The major tree branches constitute 7 modules labeled with different colors. **(C)** Proportion of genes in each module in response to herbicide application. **(D–F)** Co-expression network of module yellow, module red and module brown, respectively. The types of each transcription factor family are shown in the figure legend. **(G)** Heatmap of GO enrichment analysis of genes in module yellow (upper), red (medium) and brown (bottom), respectively.

To further understand the molecular mechanisms between gene expression patterns and the herbicide response, we performed a weighted gene co-expression network analysis (WGCNA) using 10,467 genes expressed over a 24-period on 48 h time-scale samples. We found that samples obtained during the day could be classified into two clusters, based on whether they were obtained in the morning or afternoon, while samples obtained at night were all clustered together (**Figure 4A**). These genes were classified into 7 co-expression modules *via* WGCNA (**Figure 4B**) and the turquoise and black modules had the most (4,480) and least (59) number of genes, respectively. The blue, brown, green, red, and yellow modules had 2,557, 1,777, 488, 170, and 862 genes, respectively (**Table S8**).

Next, we found that the highest percentages of both herbicide-responsive genes and herbicide safener-responsive genes could be observed in the module black (**Figure 4C**) and peaked in abundance at ZT18 (**Figure S3**). Interestingly, it was almost always the induced genes that were in this module except S-Metolachlor application. Besides the module black, the second-most percentage of glyphosate-induced genes occurred in the module brown, and their peak abundance was reached before midnight (ZT15-ZT18 and ZT39-ZT42) (**Figure 4C**). However, the highest percentage of glyphosate-suppressed genes occurred in the module green and red (**Figure 4C**), and their peak abundance was reached in the morning ((ZT3-ZT6, ZT27-ZT30) (module green) and ZT30 (module red)) (**Figure S3**). Another herbicide, S-Metolachlor, which induced genes were most abundant in module red and the most abundant suppressed genes were almost same in module green, red and turquoise in addition to the black module (**Figure 4C**). For herbicide safener, the second-most percentage of fenclorim-induced genes were the module yellow (**Figure 4C**), which reached peak abundance at dawn (ZT0, ZT3, ZT24, ZT27 and ZT48) (**Figure S3**) and the module red had the highest percentage of fenclorim-suppressed genes, and these findings were similar to those for glyphosate-suppressed genes (**Figure 4C**), which reached their peak abundance at ZT30 (**Figure S3**). In addition to the module black, the percentage of metcamifen-induced genes and GA3-induced genes were second highest in the module red. And ga3-surppressed genes were second highest in the module Green (**Figure 4C**).

Interestingly, we found that the module yellow (phased in ZT0-3; ZT24-27), module red (phased in 6-9; ZT30), and module brown (phased in ZT15-18; ZT 39-42) contained specific genes that were highly expressed in the morning, afternoon, and evening, respectively (**Figure S3**). In the module yellow, 133, 4, 18, 27 and 28 genes responded to glyphosate, S-Metolachlor, fenclorim, metcamifen and GA3 application, respectively. In addition, we identified rice central oscillator gene *OsCCA1* and 48 transcription factors in this module. Among the transcription factor families, bZIP, GRAS, MYB, bHLH, ERF, and Nin-like families were the most abundant (larger than 3) (**Figure 4D**). In the module red, 28, 2, 5, 5 and 7 genes showed a response to glyphosate, S-Metolachlor, fenclorim, metcamifen and GA3 application, respectively. In addition, we identified rice central oscillator gene *OsPRR37* and 7 transcription factors, including those from the bHLH, C3H, CO-like, LSD, and NAC transcription factor families (**Figure 4E**). In the module brown, 207, 8, 26, 26 and 44 genes responded to glyphosate, S-Metolachlor, fenclorim, metcamifen and GA3 application, respectively. In addition, we identified rice central oscillator genes, such as *OsZTL2* and *OsLWD2*, and 80 transcription factors in this module. Among the transcription factor families, the CO-like, B3, bHLH, bZIP, FAR1, MYB, and WRKY families occurred most abundantly (larger than 5) (**Figure 4F**). Further, GO enrichment analysis of genes in the three modules revealed that the genes in the morning-phased module (the module yellow) are mainly involved in transmembrane transport, the genes in the afternoon-phased module (the module red) are mainly involved in photosynthesis-related processes, while the genes in the evening-phased module (the module brown) are involved in recognition of pollen, protein phosphorylation and vitamin metabolic process (**Figure 4G**).

### 2.4 *OsCCA1* directly regulates a range of herbicide-responsive genes and certain herbicide-resistance genes

*OsCCA1* is a MYB transcription factor and one of the central oscillator genes in the rice circadian clock. It has been shown that *OsCCA1* could regulate several biological processes associated with rice growth and development (Wang et al., 2020; Sun et al., 2021; Wei et al., 2022). In addition, it has been previously shown that the overexpression of *CCA1* in *Arabidopsis* leads to a disruption in the rhythmic effectiveness of glyphosate application (Belbin et al., 2019). To explore whether *OsCCA1* directly affects the response to herbicides and herbicide safeners, we used DNA affinity purification sequencing (DAP-seq) of *OsCCA1* (Wei et al., 2022). Among the identified binding peaks, 4,078 were located within regions that were 1 kb upstream of the ATG codon and contained 4,031 downstream genes that may be directly regulated.

**Figure 5:**
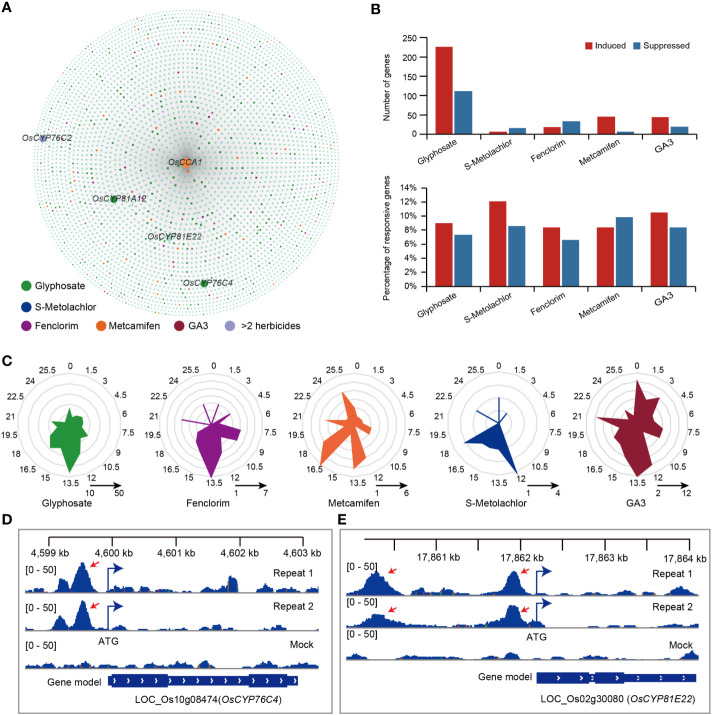
The crosstalk of *OsCCA1* target genes and herbicide-responsive genes. **(A)** *OsCCA1* and its target genes (*OsCCA1* has a binding peak within 1000 bp upstream of the start codon of this gene.) association network, the closer to *OsCCA1* gene indicates that the DAP-seq peak is closer to the promoter of the gene. **(B)** The statistics of genes induced or suppressed by herbicide in *OsCCA1* target genes. upper: the number of herbicide-responsive genes in *OsCCA1* target gene sets. bottom: the proportion of *OsCCA1* targeted herbicide-responsive genes in total herbicide-responsive genes. **(C)** Phase distribution of herbicide-responsive *OsCCA1* target genes. **(D)** The binding peaks (repeats 1 and 2) and negative control (mock) of *OsCCA1* in the promoter (–549 bp) of *OsCYP76C4* by DAP-seq. The [0–50] shows the scale bar of binding peak that refers to the height of the peak. **(E)** The binding peaks (repeats 1 and 2) and negative control (mock) of *OsCCA1* in the promoter (–276 bp) of *OsCYP81E22* by DAP-seq. The [0–50] shows the scale bar of binding peak that refers to the height of the peak.

Among the genes that were possibly regulated by *OsCCA1*, we identified 339 (8.40% in glyphosate-responsive genes), 22 (9.24% in S-Metolachlor-responsive genes), 53 (7.08% in fenclorim-responsive genes), 53 (8.54% in metcamifen-responsive genes) and 63 (9.92% in ga3-responsive genes) genes that showed a response to glyphosate (OsCCA1-Gly), S-Metolachlor (OsCCA1-SMet), fenclorim (OsCCA1-Fen), metcamifen (OsCCA1-Met) and GA3 (OsCCA1-ga3) application, respectively (**Figures 5A, B**). Of these, 63 genes responded to at least two herbicides (**Figure 5A**). However, only 7% (4,031/57,585) of genes at the genome-wide level may be directly regulated by *OsCCA1*, suggesting that *OsCCA1* may be involved in the response process to herbicides except fenclorim.

Next, we analyzed the phase distribution of these genes and found that most *OsCCA1*-regulated genes associated with a response to herbicides reached peak abundance levels in the evening. Among these, OsCCA1-Gly, OsCCA1-Fen and OsCCA1-ga3 mainly reached peak expression levels at ZT13.5, OsCCA1-Met mainly reached peak expression levels at ZT16.5 and OsCCA1-SMet mainly enriched in ZT12 (**Figure 5C**). The most herbicide-responsive genes regulated by *OsCCA1* all reached peak expression mainly at night, suggesting that *OsCCA1*-regulated processes associated with responsiveness to herbicides may play a greater role at night.

Notably, we identified four herbicide resistance genes may be directly regulated by *OsCCA1*, including *OsCYP81A12*, which homologous in *Echinochloa phyllopogon* could confer bensulfuron-methyl and penoxsulam resistance (Iwakami et al., 2014), *OsCYP81E22* which homologs in soybean to confer bentazon resistance (Kato et al., 2020), *OsCYP76C2* and *OsCYP76C4* which homologs in *Arabidopsis thaliana* to confer Monoterpenols and Phenylurea resistance (Hofer et al., 2014) (**Figure 5A**). We identified significant binding peaks at 295 bp, 863 bp and 549 bp before the start codon of *OsCYP81A12* (**Figure S4**), *OsCYP76C2* (**Figure S5**) and *OsCYP76C4* (**Figure 5D**), respectively. In addition, we identified two significant binding peaks at a location 276 bp upstream of the start codon of *OsCYP81E22* (**Figure 5E**). Notably, we also identified a significant binding peak located 4,551 bp upstream of the start codon of *OsABCC8*, which facilitated the translocation of glyphosate into the vesicles and protected plants from glyphosate toxicity (Pan et al., 2021) (**Figure S6**).

Next, we analyzed the response of these herbicide resistance genes, which may be directly regulated by *OsCCA1*, to herbicide and herbicide safener applications. We found that *OsCYP81A12* (log_2_foldchange = 2.95, FDR = 0.0067) was significantly induced upon glyphosate application, while *OsCYP76C2* (log_2_foldchange = −3.07, FDR = 0.045 and *OsCYP76C4* (log_2_foldchange = −1.15, FDR = 1.69e-14) were significantly suppressed upon glyphosate application. And the application of metcamifen, significantly induced the expression of *OsCYP76C2* (log_2_foldchange = 5.15, FDR = 2.41e-43) (**Table S9**).

### 2.5 Effect of herbicide application on rice central oscillator

**Figure 6:**
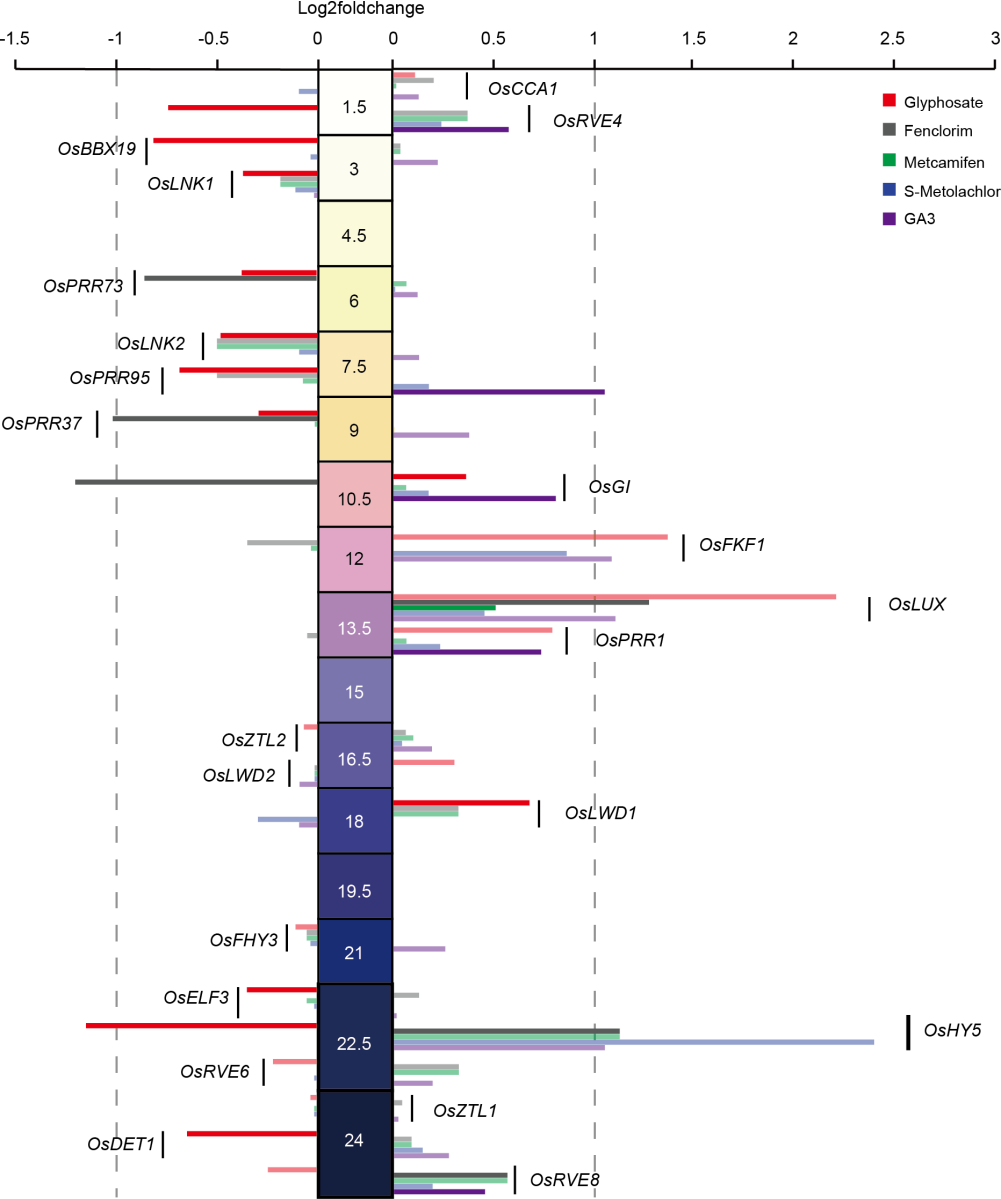
Effects of herbicide application on rice central circadian oscillator. The middle values indicate the phase (ZT) of genes. Opaque columns indicate that a significance level has been achieved (FDR<0.05).

To investigate whether the application of herbicides disrupted the central oscillator of the circadian clock in rice, we extracted central oscillator genes associated with the circadian clock in rice (Hayama and Coupland, 2004; Song et al., 2010; Filichkin et al., 2011; Greenham and McClung, 2015; Steed et al., 2021) and analyzed their expression phased in LY2186 and the changes in their expression levels before and after herbicide application (**Figure 6**).

For herbicides application, we found that only *OsHY5* (log_2_foldchange = −1.16, p-value = 1.05e-11) was significantly suppressed upon glyphosate application and no central oscillator gene significantly responds to S-Metolachlor application. For herbicide safeners application, we found four genes were significantly affected by fenclorim application, with *OsLUX* (log_2_foldchange = 1.27, p-value = 0.0048) and *OsHY5* (log_2_foldchange = 1.12, p-value = 0.00022) being significantly induced, and *OsPRR37* (log_2_foldchange = −1.04, p-value = 0.0014) and *OsGI* (log_2_foldchange = −1.22, p-value = 4.09e-06) being significantly suppressed. And *OsPRR95* was significantly induced by GA3 application. However, no central oscillator genes were significantly affected by the application of metcamifen (**Figure 6**). This suggests that central oscillator genes respond differently to herbicides.

## 3 Discussion

Previous studies have demonstrated that the effectiveness of herbicides may vary depending on the time of day they are applied. Although numerous exogenous environmental factors in the field could influence this process, time remains the second-most influential factor after ambient temperature (Martinson et al., 2002; Miller et al., 2003). It has also been recently shown that the overexpression of the *Arabidopsis thaliana* central oscillator genes *CCA1* and *TOC1* would disrupt the rhythmic effectiveness of glyphosate (Belbin et al., 2019). Nevertheless, the crosstalk between the circadian clock and the response to herbicides remains largely unknown. A previous study found the oscillation phases of rice metabolites were enriched in the afternoon and late-night (Zhou et al., 2022). Which differed from those reported on the relatively continuous peak phase of rhythmic metabolites in animals (Krishnaiah et al., 2017). Interestingly, we found that the accumulation of 19 CRMs, including eight amino acids, three carbohydrates, three Organic acids & its derivates, three Nucleotide & its derivates, a lipid and a Vitamin, could be response to pesticide application (**Figure 2A**). They reached peak levels of metabolic abundance mainly at ZT2 or ZT14-ZT18 (**Figure 2B**). Interestingly, most of the pesticide-responsive metabolites could respond to the herbicide (**Figure 2C**), which suggests that circadian rhythms are likely to influence the response process of rice to herbicides.

Our findings showed that approximately 30% of rice genes exhibit circadian expression, and these findings are similar to those for *Arabidopsis* (Covington et al., 2008). Nearly 20% and 10% of genes in rice were circadian and conserved circadian genes, respectively (**Figure 3A**). And the function of rhythmic herbicide response genes differed when different herbicides were applied. This suggests that circadian rhythms may differ in response processes to different herbicides. However, this study was only able to explore the effect of genes with circadian rhythms when subjected to herbicide application, but the dataset was unable to explore the differential response to rice produced by herbicide application at different time points, which needs to be explored in future study.

We conducted the hierarchical clustering of temporal transcriptome samples and found that they could be divided into three categories: morning, afternoon, and evening (**Figure 4A**). This suggests that samples obtained during the day could be divided into two groups, may be based on differences in light intensity, temperature, humidity, and other environmental factors in the morning and afternoon, but all the samples obtained at night were assigned to one group. Among these co-expression modules, we found three modules, each of which contained the central circadian clock oscillator genes *OsCCA1*, *OsPRR37*, and *OsZTL2*, which represented sets of genes that were specifically expressed at high levels in the morning, afternoon, and evening, respectively (**Figures 4D–F**). In addition, we found that the three modules drive different functions. The morning-phased module is mainly involved in the transmembrane transport process, the afternoon-phased module is mainly involved in the photosynthesis-related process, and the evening-phased module is mainly involved in the pollen recognition process, protein phosphorylation process and vitamin metabolic process (**Figure 4G**).

In *Arabidopsis thaliana*, *CCA1* conferred heterosis for defense against bacteria in hybrids without growth vigor penalties and significantly enhance the growth heterosis of hybrids infected with pathogens (Yang et al., 2021). A previous study has shown that the overexpression of *CCA1* in *Arabidopsis* leads to the disruption of the rhythmic effectiveness of glyphosate application (Belbin et al., 2019). *OsCCA1*, its homolog in rice, is also able to participate in the regulation of many growth and development processes (Wang et al., 2020; Sun et al., 2021; Wei et al., 2022). We have identified 4,031 genes that *OsCCA1* might directly regulate using the DAP-seq data of *OsCCA1*, and identified several genes that exhibited a response to herbicide application (**Figure 5A**) Notably, we identified significant *OsCCA1* binding peaks in the promoter regions of the four homologous herbicide resistance genes in rice (**Figures 5D, E**). And a significant binding peak was also identified at a location that was 4,551 bp upstream of the start codon of the rice glyphosate resistance gene *OsABCC8* (**Figure S6**). This suggests that *OsCCA1* may be directly involved in regulating the response and resistance to herbicides.

## 4 Conclusion

In this study, we have revealed the crosstalk between rice circadian clock and herbicide response processes in epigenomics, transcriptomics and metabolomics level. In addition, four homologs of known herbicide resistance genes in rice including *OsCYP81A12*, *OsCYP76C2*, *OsCYP76C4* and *OsCYP81E22* were found to may be directly regulated by the central oscillator gene *OsCCA1*. Finally, we found that the central oscillator genes respond differently to herbicides. We believe this research will provide a theoretical reference for discovering rhythmic herbicide targets and thus developing new herbicides or breeding herbicide-resistant crops.

## 5 Materials and methods

### 5.1 Transcriptome analysis

The RNA sequencing reads were removed of adapters and trimmed for low-quality bases using fastp (Chen et al., 2018) (version 0.21.0). Clean reads were mapped to the rice genome (MSU-release 7) (Kawahara et al., 2013) using HISAT2 (Kim et al., 2015) (version 2.1.0) with default parameters. The JTK_CYCLE (Hughes et al., 2010) was used to identify circadian genes. The JTK_CYCLE algorithm is an available computational R script from https://openwetware.org/wiki/HughesLab: JTK_Cycle. We applied the JTK_CYCLE algorithm to estimate the period length, phase, and amplitude for the genes. Only genes with a (P-value < 0.05 and period length = 24) were considered circadian genes.

### 5.2 Co-expression network construction

The Stringtie software (version 1.3.6) (Pertea et al., 2015) was used to calculate the TPM value of genes and the R package WGCNA (Langfelder and Horvath, 2008) were used to identify co-expression modules based on the TPM data.

### 5.3 Analysis of differential expressed genes

The software featurecount (version 1.6.0) (Liao et al., 2014) was used to calculate the raw count of genes and the R package DESeq2 (Love et al., 2014) was used to identify differentially expressed genes. Log2foldchange > 1 or < -1 and adjusted p-values (FDR) < 0.05 were defined as differential expressed genes.

### 5.4 Functional annotation and classification

Gene Ontology (GO, http://www.geneontology.org/) enrichment analysis of the genes was using R package topGO (Alexa and Rahnenfuhrer, 2010).

### 5.5 DAP-seq data analysis

DAP-seq reads were aligned to the reference genome using Bowtie2 (version 2.4.2)(Langmead and Salzberg, 2012). The software MACS2 (version 2.2.7.1) was used to callpeak (Zhang et al., 2008) and IDR (version 2.0.2) software (Li et al., 2011) was used to merge the peaks of the two biological replicates with P<0.05. The bound peaks were annotated using Homer software (Heinz et al., 2010).

### 5.6 Identification of rhythmic agrochemical-responsive metabolites

We used the lists of metabolites that herbicide-induced or suppressed (Liu and Zhu, 2020), the list of circadian rhythmic metabolites (Zhou et al., 2022) and our metabolomic data about s-metolachlor and GA3 application. We found 19 metabolites that not only had stable circadian rhythms and were able to respond to pesticide application. Each of these metabolites was also assigned to bins of 2 h according to its circadian phase.

### 5.7 Identification of the rice central oscillator genes

Sequences mentioned in this article can be download from Rice Genome Annotation Project Database (http://rice.plantbiology.msu.edu/) under the following accession numbers: *OsCCA1*, LOC_Os08g06110; *OsPRR1*, LOC_Os02g40510; *OsPRR95*, LOC_Os09g36220; *OsPRR37*, LOC_Os07g49460; *OsPRR73*, LOC_Os03g17570; *OsLUX*, LOC_Os01g74020; *OsELF3*, LOC_Os06g05060; *OsZTL1*, LOC_Os02g05700; *OsZTL2*, LOC_Os06g47890; *OsFKF1*, LOC_Os11g34460; *OsGI*, LOC_Os01g08700. Other genes were derived from blast with Arabidopsis genes: *OsBBX19*, LOC_Os09g35880; *OsDET1*, LOC_Os01g01484; *OsFHY3*, LOC_Os02g39540; *OsHY5*, LOC_Os02g10860; *OsLWD1*, LOC_Os02g45810; *OsLWD2*, LOC_Os02g32430; *OsLNK1*, LOC_Os03g27019; *OsLNK2*, LOC_Os01g31360; *OsRVE4*, LOC_Os02g45670; *OsRVE6*, LOC_Os06g45840; *OsRVE8*, LOC_Os06g01670.

### 5.8 metabolite extraction

Biological samples are freeze-dried by vacuum freeze-dryer (Scientz-100F). The freeze-dried sample was crushed using a mixer mill (MM 400, Retsch) with a zirconia bead for 1.5 min at 30 Hz. Dissolve 100 mg of lyophilized powder with 1.2 mL 70% methanol solution, vortex 30 seconds every 30 minutes for 6 times in total, place the sample in a refrigerator at 4°C overnight. Following centrifugation at 12000 rpm for 10 min, the extracts were filtrated (SCAA-104, 0.22 μm pore size; ANPEL, Shanghai, China, http://www.anpel.com.cn/) before UPLC-MS/MS analysis.

### 5.9 UPLC-MS/MS conditions and qualitative

The sample extracts were analyzed using an UPLC-ESI-MS/MS system (UPLC, SHIMADZU Nexera X2, www.shimadzu.com.cn/; MS, Applied Biosystems 4500 Q TRAP, www.appliedbiosystems.com.cn/). The analytical conditions were as follows, UPLC: column, Agilent SB-C18 (1.8 μm, 2.1 mm * 100 mm); The mobile phase was consisted of solvent A, pure water with 0.1% formic acid, and solvent B, acetonitrile with 0.1% formic acid. Sample measurements were performed with a gradient program that employed the starting conditions of 95% A, 5% B. Within 9 min, a linear gradient to 5% A, 95% B was programmed, and a composition of 5% A, 95% B was kept for 1 min. Subsequently, a composition of 95% A, 5.0% B was adjusted within 1.1 min and kept for 2.9 min. The flow velocity was set as 0.35 mL per minute; The column oven was set to 40°C; The injection volume was 4 μL. The effluent was alternatively connected to an ESI-triple quadrupole-linear ion trap (QTRAP)-MS. LIT and triple quadrupole (QQQ) scans were acquired on a triple quadrupole-linear ion trap mass spectrometer (Q TRAP), AB4500 Q TRAP UPLC/MS/MS System, equipped with an ESI Turbo Ion-Spray interface, operating in positive and negative ion mode and controlled by Analyst 1.6.3 software (AB Sciex). The ESI source operation parameters were as follows: ion source, turbo spray; source temperature 550°C; ion spray voltage (IS) 5500 V (positive ion mode)/-4500 V (negative ion mode); ion source gas I (GSI), gas II(GSII), curtain gas (CUR) was set at 50, 60, and 25.0 psi, respectively; the collision-activated dissociation (CAD) was high. Instrument tuning and mass calibration were performed with 10 and 100 μmol/L polypropylene glycol solutions in QQQ and LIT modes, respectively. QQQ scans were acquired as MRM experiments with collision gas (nitrogen) set to medium. DP and CE for individual MRM transitions was done with further DP and CE optimization. A specific set of MRM transitions were monitored for each period according to the metabolites eluted within this period. Metabolite data were log2-transformed for statistical analysis to improve normality and were normalized.

## Data availability statement

The datasets presented in this study can be found in online repositories. The names of the repository/repositories and accession number(s) can be found in the article/**Supplementary Material**.

## Author contributions

LFW, TL, LB., and KC. designed the study. KC and XS performed the analysis. KC, HY, and YP wrote the manuscript. KC, LFW, LMW, and ZZ revised the manuscript. All authors contributed to the article and approved the submitted version.
