# Supplementary material for: Multi-omics analyses reveal the crosstalk between the circadian clock and the response to herbicide application in Oryza sativa
Source: Front Plant Sci. 2023 Mar 24;14:1155258. doi: 10.3389/fpls.2023.1155258 (PMC10080033; doi:10.3389/fpls.2023.1155258)
Supplement: Supplementary file 1 [file Image_1.pdf]

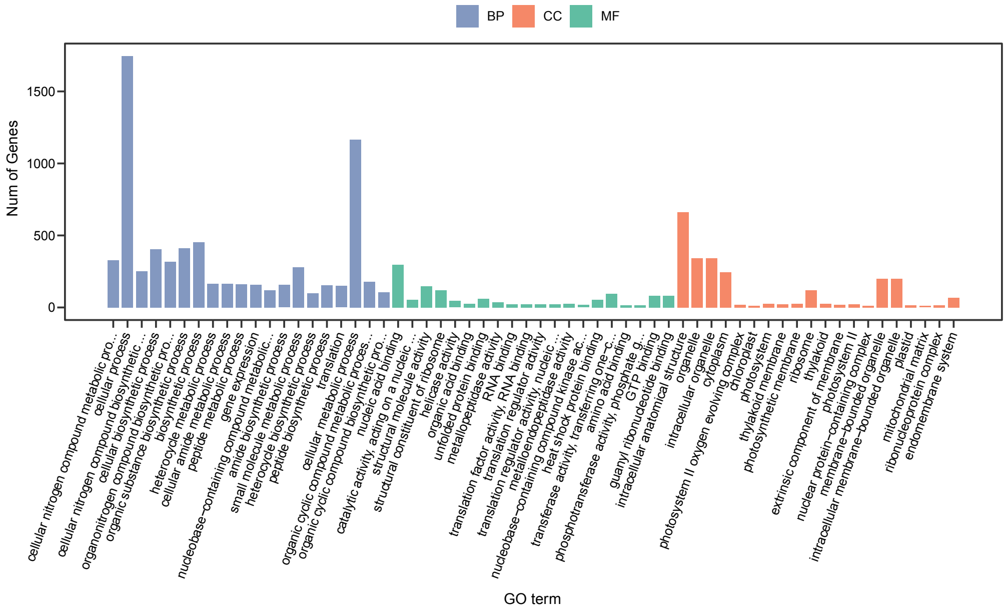

Supplementary Figure 1. GO enrichment analysis of conserved circadian genes.

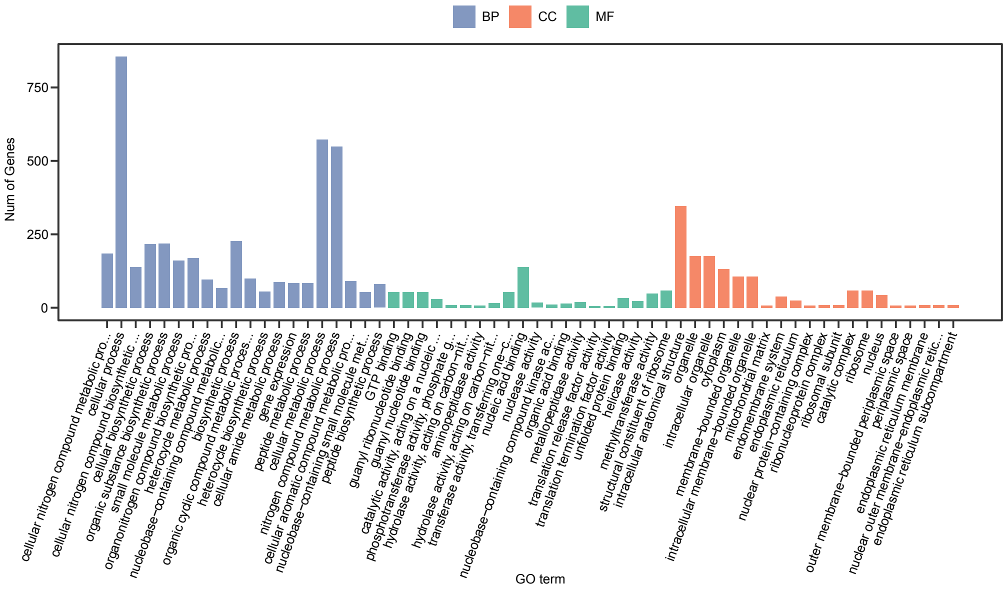

Supplementary Figure 2. GO enrichment analysis of extremely conserved circadian genes.

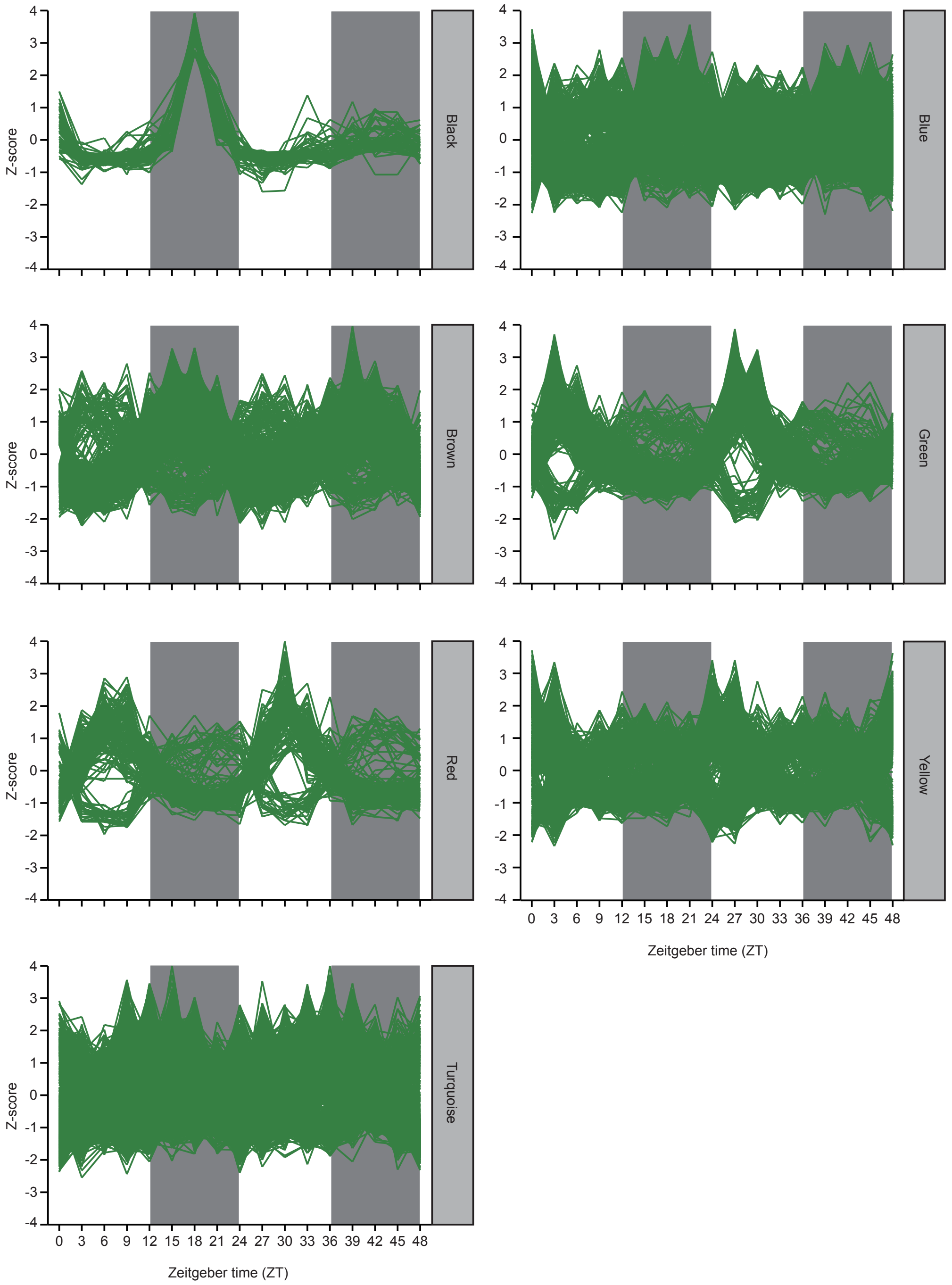

Supplementary Figure 3. Z-score plots showing expression profiles of genes in each module. Grey shading indicates subjective night.

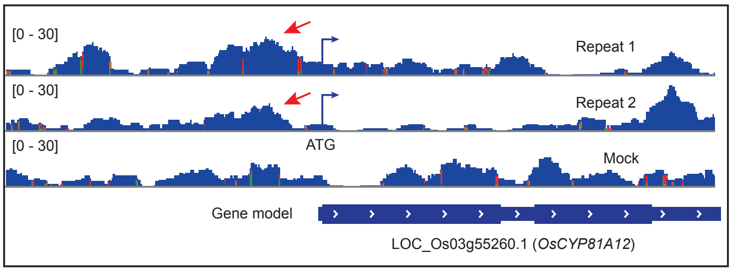

Supplementary Figure 4. The binding peaks (repeats 1 and 2) and negative control (mock) of *OsCCA1* in the promoter (–295 bp) of *OsCYP81A12* by DAP-seq. The [0–30] shows the scale bar of binding peak that refers to the height of the peak.

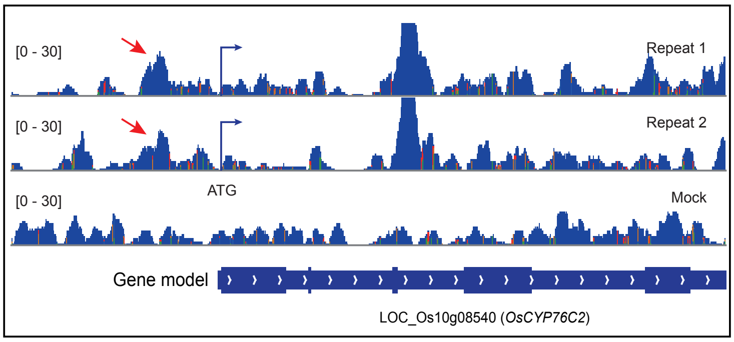

Supplementary Figure 5. The binding peaks (repeats 1 and 2) and negative control (mock) of *OsCCA1* in the promoter (-863 and -549 bp) of *OsCYP76C2* by DAP-seq. The [0–30] shows the scale bar of binding peak that refers to the height of the peak.

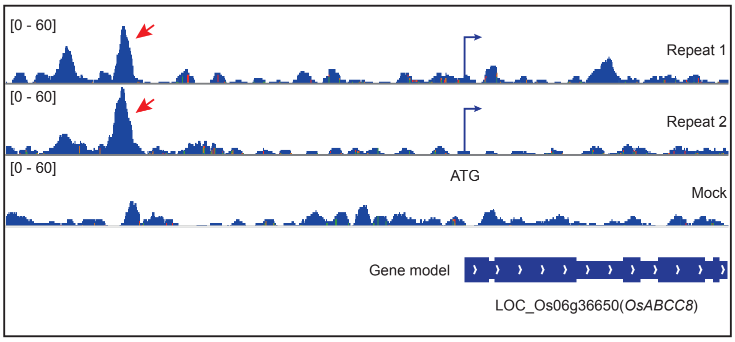

Supplementary Figure 6. The binding peaks (repeats 1 and 2) and negative control (mock) of *OsCCA1* in the upstream (–4551 bp) of *OsABCC8* by DAP-seq. The [0–60] shows the scale bar of binding peak that refers to the height of the peak.
